# Multifaceted Biodiversity Patterns and Influencing Factors of *Lucanus* Stag Beetles (Coleoptera, Lucanidae) in China

**DOI:** 10.1002/ece3.71954

**Published:** 2025-08-12

**Authors:** Ya Xin Wang, Ya Gang Shen, Xue Li Zhu, Xia Wan

**Affiliations:** ^1^ School of Resources and Environmental Engineering Anhui University Hefei China

**Keywords:** environmental filtering, historical climate, *Lucanus*, multifaceted biodiversity, spatial analyses, topographical heterogeneity

## Abstract

As keystone decomposers in forest ecosystems, stag beetles occupy unique ecological niches within forest carbon and nitrogen cycles. While previous studies have predominantly focused on taxonomic inventories of this group, we present a multidimensional assessment integrating taxonomic, phylogenetic, and functional diversity patterns across the model genus *Lucanus* of stag beetles in China. Using random forest analyses, we identify key environmental factors influencing these biodiversity dimensions—contemporary climate factors, habitat heterogeneity, and paleoclimatic fluctuations. Our results show that maximum species richness and phylogenetic diversity (Faith's PD) emerge in southwest mountain ranges (Hengduan and Gaoligong Mountains), primarily regulated by annual temperature range constrictions. This region also retains older lineages and shows phylogenetic dispersion, while South China and Taiwan exhibit more recent differentiation and phylogenetic aggregation, primarily influenced by precipitation and temperature anomalies. Functional richness and functional dispersion are higher in the southeastern region, whereas the southwest displays greater functional evenness and functional divergence, reflecting stronger environmental filtering. Functional diversity indicators are mainly driven by precipitation. These multidimensional diversity patterns exhibit geographical decoupling, with mountain ecosystems consistently serving as biodiversity arks. Our findings establish an integrative framework for insect conservation in Asian forest ecosystems, emphasizing the need for topography‐sensitive management strategies.

## Graphical abstract

As keystone indicators of forest integrity, stag beetles (genus *Lucanus*) in China reveal novel multidimensional patterns integrating taxonomic, phylogenetic, and functional diversity. Our integrative framework elucidates the interactive effects of contemporary environmental gradients and historical climatic oscillations on these biodiversity dimensions, advancing evolutionary‐informed conservation strategies for forest arthropods.

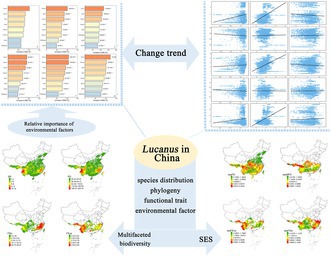

## 1 Introduction

Due to the differentiation of global environmental conditions, the distribution of biodiversity across different regions exhibits highly uneven characteristics (Rangel et al. 2018). On the one hand, environmental conditions determine the availability of resources and define corresponding ecological niche spaces (Riano and Moulatlet 2022); on the other hand, historical processes—such as speciation, extinction, and dispersal—leave enduring evolutionary imprints on the composition of biological communities, manifested as phylogenetic clustering or dispersion patterns (Kissling et al. 2012; Qian et al. 2024). Additionally, environmental filtering and biotic interactions (such as competition or mutualistic relationships) can drive the convergence or divergence of functional traits, further shaping the spatial structure of biodiversity (Mouchet et al. 2010; Riano and Moulatlet 2022). Against the backdrop of an intensifying global biodiversity crisis and limited conservation resources, studying the spatial distribution of species is crucial for understanding the origins of biodiversity and identifying effective conservation priorities.

Biodiversity typically encompasses three dimensions: species diversity (taxonomy), functional diversity, and phylogenetic diversity (Lyashevska and Farnsworth 2012). Previous studies have focused on the single dimension of species diversity, but this dimension alone is not sufficient to fully explain the overall structure of an ecosystem or biome. This underscores the value of integrating multiple dimensions of biodiversity perspectives (Freitas et al. 2021; Montaño‐Centellas et al. 2023). Unlike species diversity, phylogenetic and functional diversity focus on species traits, particularly in terms of evolutionary history and ecological function. Phylogenetic diversity captures the evolutionary relationships between species, offering insights into long‐term adaptation and community evolution (Hu et al. 2021). Functional diversity reflects the variation in species traits and, when combined with environmental factors, helps explain species turnover and resource utilization strategies within communities (Villéger et al. 2008; Fountain‐Jones et al. 2015). The inconsistency among different diversity indices suggests that conclusions drawn from a single diversity index may not accurately describe the spatial variation of biodiversity. Therefore, integrating multiple dimensions in biodiversity research helps deepen our understanding of the mechanisms shaping species distributional patterns, thus revealing the ecological and evolutionary processes behind diversity patterns.

Contemporary climate, habitat heterogeneity, and historical climate change are widely recognized as key determinants of species diversity, although their relative contributions vary across regions and taxonomic groups (Liu et al. 2019; Song et al. 2024a, 2024b). The average temperature during the coldest season is the primary factor shaping woody plant species richness in China (Wang et al. 2011). Similarly, both ancient climate and contemporary conditions drive the evolutionary and functional diversity of global raptors (Montaño‐Centellas et al. 2023), while habitat heterogeneity and long‐term climate change effectively predict the spatial variation in the richness of narrow‐range species (Liu et al. 2022). Recent studies highlight the multifactorial drivers of beetle diversity. For Neotropical dung beetles, species richness is driven by the combined effects of productivity, resource heterogeneity, and spatially structured factors, with abundance mediating energy relationships (Pessôa et al. 2021). Moctezuma et al. in their study of coprophilic scarab guilds within Mexico's transition zone demonstrated multifactorial structuring: species richness correlated with productivity, diversity reflected energy tradeoffs, and community composition was driven by habitat heterogeneity (Moctezuma et al. 2024). This reinforces the necessity of integrated hypothesis frameworks. These findings underscore the multifaceted and context‐dependent nature of biodiversity distribution drivers; exploring the factors driving those diversity patterns provides a stronger scientific foundation for optimizing conservation strategies.

Lucanidae (Coleoptera: Scarabaeoidea) stag beetles are an important saprophytic group with a complex life history. During the larval stage, stag beetles primarily feed on decaying wood, while the images are known to feed on tree sap, flowers, and rotting fruit. As a result, stag beetles have a unique ecological niche in the carbon cycle of forest ecosystems (Tanahashi and Kubota 2013; Songvorawit et al. 2017). Additionally, due to their high sensitivity to changes in habitat temperature, humidity, and human disturbances, stag beetles are of significant importance as ecological indicators for forest environments (Harvey et al. 2011; Lachat et al. 2012). Research on distributional patterns can help to reveal how stag beetles respond to environmental changes. However, due to the lack of distributional data, relevant studies are currently scarce and have focused primarily on species diversity (Chen et al. 2020). Therefore, investigating the multidimensional biodiversity patterns (including taxonomic, functional, and phylogenetic diversity) and distribution of *Lucanus* Scopoli stag beetles in China will not only address gaps in current ecological knowledge but also provide scientific foundations for forest ecosystem monitoring and conservation.

China has a vast territory, diverse landscapes, and spans multiple climatic zones (Tang et al. 2006). According to statistics, the known number of stag beetle species in China accounts for approximately 18.6% of the total global species (Bánki et al. 2025; Huang and Chen 2010, 2013, 2017). With the impact of global climate change and human activities, the habitat of stag beetles is facing challenges (Harvey et al. 2023). In order to better understand the impact of these changes on the diversity of stag beetles and provide scientific support for their conservation, our study selects the genus of stag beetles *Lucanus* Scopoli in order to address the following scientific questions through the analysis of distributional data, mitochondrial gene data, and functional traits in China: (1) What are the geographic distributional patterns of *Lucanus* diversity across taxonomic, functional, and phylogenetic dimensions in China? (2) What is the relative importance of contemporary climate, habitat heterogeneity, and historical climate change in influencing the distribution of *Lucanus* diversity? (3) What are the underlying mechanisms of diversity distribution in *Lucanus*?

## 2 Materials and Methods

### 2.1 Occurrence Data and Species Distribution Models

To investigate the potential geographic distributional patterns of *Lucanus* in China, we compiled geographical distribution data from multiple available sources, including *Chinese Stag Beetles I‐III* (Huang and Chen 2010, 2013, 2017), the Global Biodiversity Information Facility (GBIF) database (http://www.gbif.org, accessed in 2022), specimen records from domestic and international universities and museums, field samples collected by our research group, relevant published literature, and data provided by the Bio‐Nica website (http://www.bio‐nica.info/home/index.html, accessed in 2022). Any doubtful distribution records were excluded from the dataset prior to the analysis. We compiled a total of 2152 collection records across 76 species and subspecies of *Lucanus* in China. Corresponding spatial visualizations, including kernel density estimation and hexagonal binning, are provided in the Supporting Information to illustrate distributional patterns and collection intensity (Figures [Link], [Link]).

To minimize sampling biases caused by uneven data collection across regions, the terrestrial map of China was divided into spatial grid cells with a spatial resolution of 20 km × 20 km. Grids with less than 50% coverage along the borders and those whose centroid coordinates fell outside of China were excluded. This resulted in 29,684 grids being retained (Xu et al. 2024). The longitude and latitude of the township administrative unit where the species is located are projected into the 20 × 20 km grid as the coordinates of the record. To ensure statistical robustness, we excluded species with fewer than five occurrence records across grids (Wang et al. 2024). Only the unique collection record of the same species is retained in the same grid, which represents the existence of the species in the region. Therefore, we use ENMTools1.4.4 (Warren et al. 2010) for processing of duplicate data within the same cell grid (20 × 20 km). Following data cleaning and calibration, 737 unique, geographically valid distribution records representing 59 species and subspecies of *Lucanus* were retained for subsequent analysis.

We downloaded 19 bioclimatic variables and SRTM elevation data (2.5 arc‐minute resolution) from the WorldClim database (http://www.worldclim.org, version 2.1, accessed in 2024). The normalized difference vegetation index (NDVI) data, downloaded from the Institute of Resources and Environmental Sciences, Chinese Academy of Sciences (http://www.resdc.cn, accessed in 2024), with a 1 km resolution, was resampled to a 2.5 arc‐minute resolution. We used these datasets, along with distribution records of 59 *Lucanus* species and subspecies, for ecological niche modeling and distributional prediction in Maxent 3.4.4 (Phillips et al. 2006), aiming to better capture the effects of incomplete sampling (Liu et al. 2018). To prevent model overfitting, we removed environmental variables with high multicollinearity (VIF ≥ 5, *r* > |0.75|). Ultimately, we retained the following variables for subsequent modeling: mean diurnal temperature range, annual temperature range, mean annual precipitation, precipitation variance, and normalized difference vegetation index. These variables were selected to ensure the robustness and reliability of the ecological niche models. Threshold selection criteria in Maxent were tailored based on the number of occurrence records: for species with 5–9 records, thresholds were set at the point where specificity equals sensitivity; for species with ≥ 10 records, the 10% training presence logistic threshold was applied to determine species presence (Dong et al. 2019). To minimize overprediction, we used SDMtoolbox 2.0 (Brown 2014) within ArcMap 10.8, applying a 100 km buffer to generate a minimum convex polygon for model calibration. We then applied a binary thresholding method to the calibrated Maxent predictions. The resulting presence/absence data were converted to latitude and longitude coordinates and spatially joined with the 20 × 20 km grid in ArcMap. This process resulted in a dataset where each grid cell corresponds to the presence/absence of each species, forming the basis for subsequent biodiversity analysis.

### 2.2 Phylogenetic Tree

To reconstruct the phylogenetic relationships of those *Lucanus* species, we utilized two mitochondrial genes (COI, 16S rDNA) as molecular markers. Due to the absence of molecular data for *Lucanus davidis* Deyrolle 1878, this species was excluded from the phylogenetic analysis. The final phylogenetic reconstruction was based on data from 58 species and subspecies of *Lucanus*, and the outgroups: *Cyclommatus albersii* Kraatz 1894 *and Cyclommatus mniszechi* Thomson 1856. The molecular data were obtained from the NCBI database and supplemented with experimental data generated through genomic DNA extraction using the DNA Blood & Tissue Kit (QIAGEN) (Zhu et al. 2023). The phylogenetic tree was constructed using BEAST v.2.6 software to generate a maximum credibility tree (MCMCtree) for the *Lucanus* genus. A strict molecular clock model was applied, with the evolutionary rates for COI and 16S rDNA set to 0.017 and 0.0054 (Zhu et al. 2023), respectively. The analysis was run for 1 billion generations, with sampling every 1000 generations. Convergence and reliability of the results were evaluated using Tracer v.1.7 (Rambaut et al. 2018), where an Effective Sample Size (ESS) greater than 200 was deemed satisfactory. TreeAnnotator v. 2.5.1 was then used to discard the first 25% of burn‐in samples and calculate the optimal tree for subsequent analysis (Bouckaert et al. 2014). The resulting phylogenetic tree, including nodal support values, is shown in Figure S3.

### 2.3 Functional Trait

We measured 10 independent morphological traits (see Table S1) across 56 male species and subspecies of *Lucanus*: body length, head width, pronotum length and width, elytra aspect ratio, front femur length, front tibia length, antenna length, eye length, and mandibular aspect ratio. The annotated diagram of functional traits, using *Lucanus brivioi* Zilioli 2003, is shown in Figure S4. These traits were selected based on their ecological relevance as documented in the literature (Fountain‐Jones et al. 2015; Hagge et al. 2021). Body shape traits influence dispersal ability, metabolic rate, and structural robustness. Locomotion traits determine flight performance and foraging efficiency, while sensory traits govern olfactory communication, visual capacity, and activity patterns. Foraging traits are linked to resource acquisition and reproductive interactions. These morphological traits, which can be accurately assessed from specimen images, reflect the ecological strategies and functional adaptations of the species. However, due to the incompleteness of specimens, we could not measure the morphological characteristics of three of these species (*Lucanus kanoi piceus* Kurosawa 1966; *Lucanus ogakii chuyunshanus* Sakaino and Yu 1993; *Lucanus ogakii ogakii* Nagai 2000).

Of the individuals measured, 1.9% had incomplete specimens, and some traits could not be measured. Given the incompleteness of trait combination data, we employed the missForest algorithm combined with phylogenetic information to impute missing data (Debastiani et al. 2021). The *missForest* package was used to handle trait data, with phylogenetic information incorporated as Phylogenetic Eigenvectors as additional predictor variables (Stekhoven and Stekhoven 2013; Santos et al. 2018). The model parameters were set to a maximum of 15 iterations and 100 trees, and imputation accuracy was assessed using the Out‐of‐Bag error. The completed dataset was then saved as a CSV file for subsequent analysis.

### 2.4 Environmental Factors

To explore the impact of environmental factors on the distributional patterns of *Lucanus* diversity in China, we considered a series of environmental variables that are widely examined in large‐scale species diversity research (Wang et al. 2011). These variables encompass a range of factors, including current climatic conditions, paleoclimatic change variables, and habitat heterogeneity. For the current environmental conditions, we downloaded 19 bioclimatic variables from the WorldClim database (http://www.worldclim.org, version 1.4, accessed in 2024) at a spatial resolution of 2.5 arc‐minutes. Paleoclimatic change variables include changes in temperature and precipitation since the last glacial maximum (LGM) (Song et al. 2024b). We downloaded the annual mean temperature and annual precipitation reconstructed for the LGM using the CCSM4 model (Gent et al. 2011) from WorldClim, at a spatial resolution of 2.5 arc‐minutes. Paleoclimatic fluctuations were quantified as the absolute differences between LGM and contemporary values of temperature and precipitation (MAT anomaly and MAP anomaly) (Song et al. 2024b; Liu et al. 2022). In the subsequent analyses, we projected these values onto a 20 × 20 km grid and calculated the mean value of each environmental variable for each grid cell. Habitat heterogeneity was quantified using elevation range (Kerr and Packer 1997; Song et al. 2024b), calculated as the difference between maximum and minimum elevations within each 20 × 20 km grid cell, based on 2.5 arc‐minute SRTM elevation data (http://www.worldclim.org, version 2.1, accessed in 2024). Additionally, the vegetation productivity was represented by the Normalized Difference Vegetation Index (NDVI), which serves as an indicator of net primary productivity and is strongly correlated with biological productivity and plant diversity in ecosystems (Dolson and Kharouba 2024).

To detect multicollinearity among the initial 24 variables, we applied the variance inflation factor (VIF < 5) and used this method to remove strong multicollinearity (*r* > |0.75|) (Yao et al. 2022). After filtering, the environmental factors retained for subsequent analyses differed slightly among diversity metrics. For species richness (SR) and Faith's phylogenetic diversity (Faith's PD), six variables were retained: annual temperature range, mean annual precipitation, precipitation variance, NDVI, temperature anomaly, precipitation anomaly, and elevation range. For other diversity indices, eight variables were retained: mean diurnal temperature range, annual temperature range, mean annual precipitation, precipitation variance, NDVI, temperature anomaly, precipitation anomaly, and elevation range. Table S2 summarizes these variables and their abbreviations.

### 2.5 Species Richness, Phylogenetic Diversity, Functional Diversity

Firstly, based on the above species‐cell matrix, we assessed the richness of *Lucanus* species within each grid cell by counting the number of unique species present within it.

Different phylogenetic structure indices reflect multiple characteristics of the phylogenetic structure. For assessing phylogenetic diversity, combining multiple indices for quantification helps to gain a more comprehensive understanding of phylogenetic structure (Qian et al. 2024). In our study, three complementary indices were calculated, including Faith's PD, MPD (mean pairwise distance), and MNTD (mean nearest taxon distance) (Montaño‐Centellas et al. 2023), to quantify the phylogenetic diversity of *Lucanus* species within grid cells using the *picante* package (Kembel et al. 2010). Faith's phylogenetic diversity (Faith's PD) measures total evolutionary differentiation, MPD reflects overall phylogenetic divergence, and MNTD highlights local variations in phylogenetic relatedness. These metrics were based on the topology generated by the phylogenetic tree and calculated.

For functional diversity, we calculated functional richness (FRic), functional evenness (FEve), functional dispersion (FDis), and functional divergence (FDiv) based on eight selected functional traits (Table S1) using the FD package (Laliberté and Legendre 2010). Functional richness represents the amount of functional space occupied by the species assemblage. Functional evenness corresponds to the distribution frequency of species abundance within the functional space. Functional dispersion measures the weighted average distance of each species from the centroid in the functional space, serving as an indicator of the functional similarity among key community members. Functional divergence reflects the disparity in organism trait values within the community, indicating niche differentiation and resource competition (Mason et al. 2005; Laliberté and Legendre 2010). Prior to analysis, all trait values were standardized to a mean of 0 and a variance of 1 to meet the prerequisites for subsequent analyses.

### 2.6 Null Model in Phylogenetic Diversity and Functional Diversity

To mitigate the influence of species richness and community size, we calculated the standardized effect size (SES) of each phylogenetic and functional diversity index by transforming the original indices into standardized deviation scores through comparison with expected values generated from randomized communities (Montaño‐Centellas et al. 2023). Using a null model that accounts for species richness, the SES values provide a quantitative measure of phylogenetic and functional trait dispersion, enabling meaningful comparisons of phylogenetic structure across communities and quantifying the phylogenetic and functional structure of *Lucanus* in China (Cadotte and Davies 2016; Montaño‐Centellas et al. 2023). The formula for calculating SES is as follows:

$$\text{SESMetric}=\frac{\mathrm{Obs}-{\text{Mean}}_{\text{null}}}{{\mathrm{SD}}_{\text{null}}}$$

In this formula, Observed represents the observed diversity value within each grid cell, while Mean_null_ and SD_null_ denote the mean and standard deviation, respectively, of the phylogenetic or functional diversity calculated from 999 randomly generated communities in each grid cell. In each grid cell, the corresponding positive and negative SES values indicate that the observed value is, respectively, higher or lower than the mean expected value, reflecting the relative dispersion or aggregation of species (Swenson 2014).

### 2.7 Environmental Predictors of *Lucanus* Diversity

To examine the relationship between species richness and environmental variables, we performed univariate generalized linear models (GLMs) with a quasi‐Poisson regression (Liu et al. 2022). We then applied linear regression analysis using the *lm* function to explore the relationship between phylogenetic diversity, functional diversity, and their SES with environmental variables.

Building on the ability of random forests to effectively capture nonlinear relationships and complex interactions between diversity metrics and environmental variables while being less influenced by input factor correlations and spatial dependencies (Cutler et al. 2007), we utilized the Random Forest model to assess the relative importance of selected environmental variables in explaining the variation in diversity metrics. The contribution of each environmental variable to the diversity metrics was evaluated using the percentage increase in mean squared error (MSE) as an importance metric. The analysis was conducted using the *randomForest* and *rfPermute* packages in R (Archer and Archer 2016), with the following parameters: importance = TRUE, ntree = 1000, and nrep = 1000. In the random forest framework, the percentage increase in MSE indicates the relative importance of an environmental variable; a higher MSE percentage corresponds to greater relative importance (Liao et al. 2024).

All analyses in this study were conducted using R v.4.3.3 (R Core Team 2024).

## 3 Results

### 3.1 Distributional Patterns of Multifaceted Diversity

**FIGURE 1:**
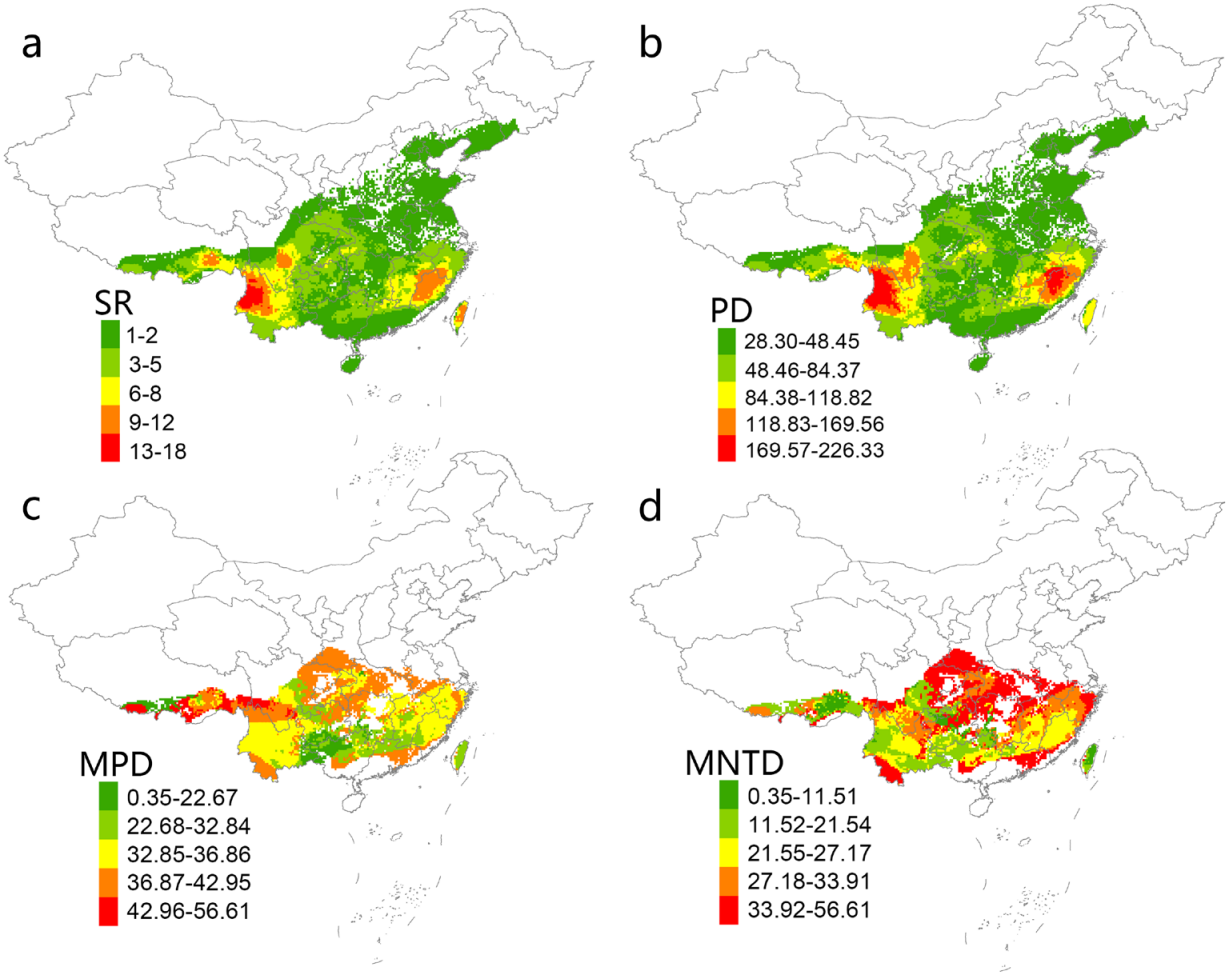
Spatial pattern of multifaceted diversity metrics of *Lucanus* beetles in China. Observed values of species richness (SR; a), Faith's phylogenetic diversity index (Faith's PD; b), mean pairwise distance (MPD; c), mean nearest taxon distance (MNTD; d).

The *Lucanus* beetles in China are primarily distributed in the southern regions, with the highest species richness concentrated in areas such as the Nu and Gaoligong Mountains within the Hengduan Mountain Range (Figure 1a). Other regions, including the Eastern Himalayas, the southwestern Hengduan Mountains, Wuyi Mountains, and the Taiwan Mountain Range, also exhibit relatively high species richness. As expected, the distributional pattern of observed Faith's PD closely mirrored that of species richness (Figure 1b). However, in regions with lower species richness, such as Central China, South China, the Sichuan Basin, and parts of southern Tibet, both MPD and MNTD were relatively high (Figure 1c,d). In contrast, MPD and MNTD values were generally lower in the northern parts of South China and the Taiwan Mountain Range (Figure 1c,d). Notably, the southeastern Tibet region exhibited higher MPD values overall; MNTD was relatively lower in this area.

**FIGURE 2:**
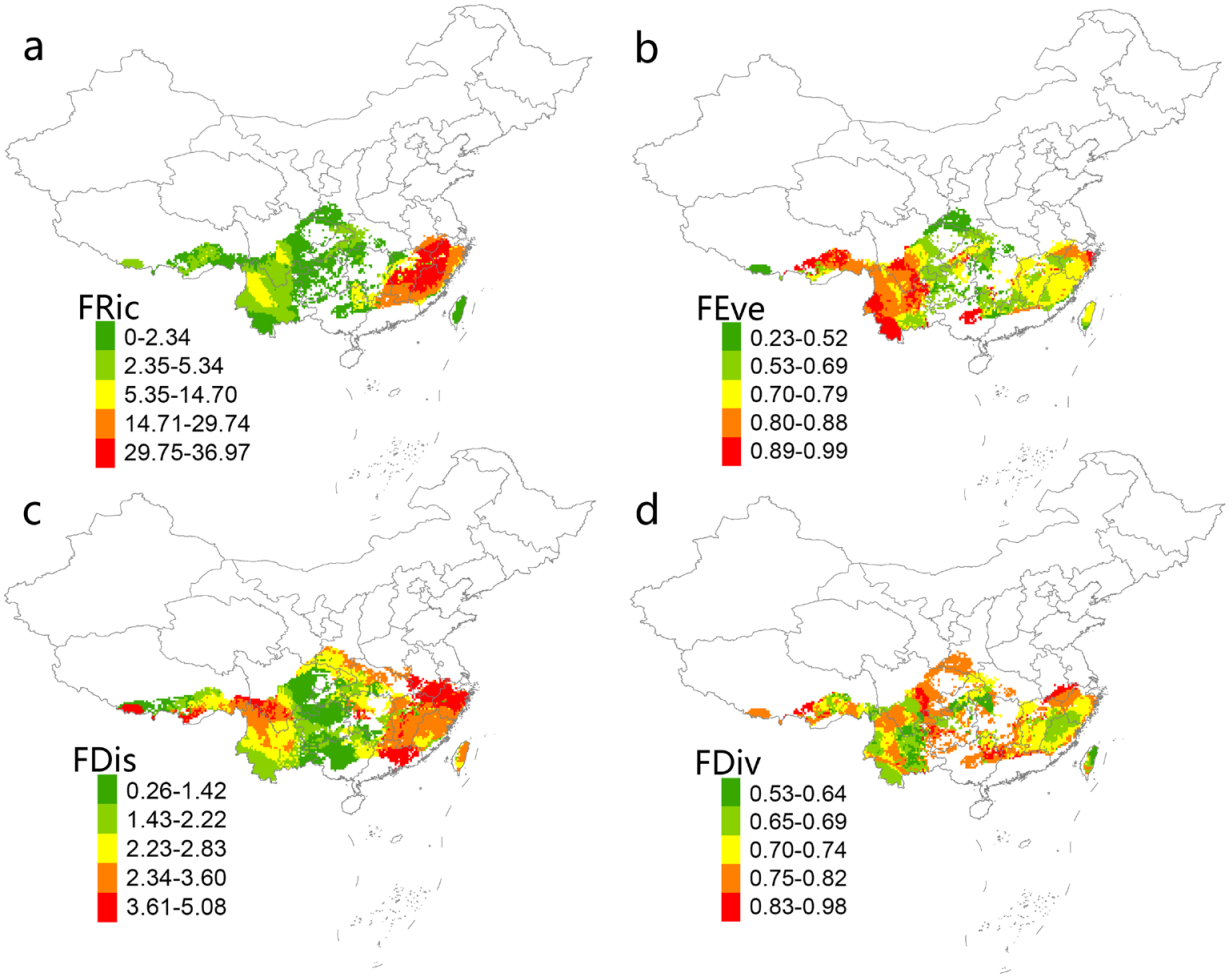
Spatial pattern of multifaceted diversity metrics of *Lucanus* beetles in China. Observed values of functional richness (FRic; a), functional evenness (FEve; b), functional dispersion (FDis, c), functional divergence (FDiv; d).

The functional diversity metrics indicated that high functional richness values were mainly concentrated in the East China region, suggesting that species in this area occupy a broad functional space with a large volume of functional space. In contrast, other regions generally showed lower functional richness values (Figure 2a). Functional dispersion was higher in East China, eastern South China, southern Tibet, and the Hengduan Mountain Range, indicating greater species differences in these areas (Figure 2c). Compared with the southeastern regions, the southwestern region exhibited overall higher functional evenness, suggesting that the available resources in ecological niche spaces were more efficiently utilized in this area (Figure 2b). The regions with high functional divergence largely coincide with the locations of the southern major mountain ranges (Figure 2d).

### 3.2 Richness‐Controlled Phylogenetic and Functional Diversity

**FIGURE 3:**
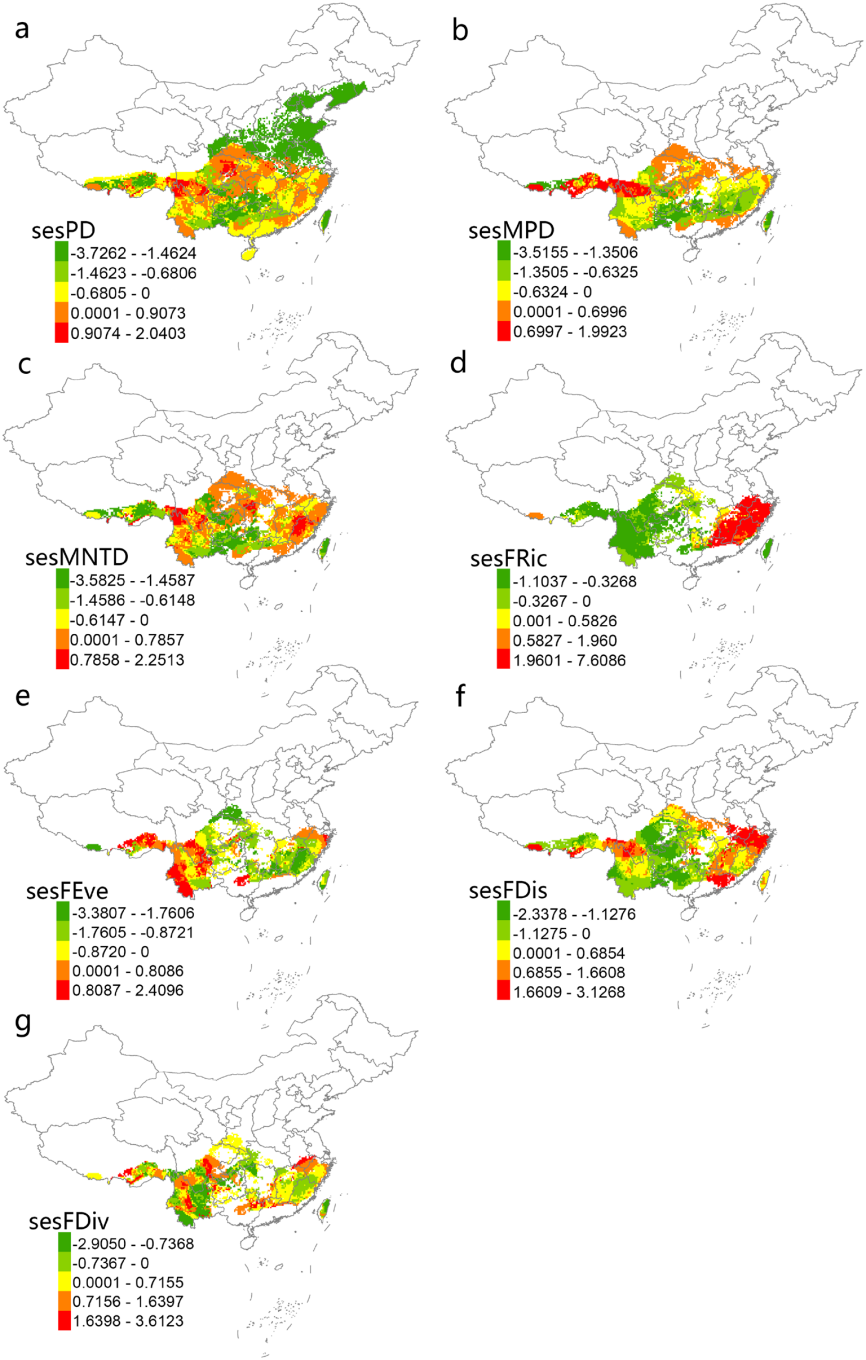
Standardized effect size (SES) of Faith's phylogenetic diversity index (Faith's PD; a), mean pairwise distance (MPD; b), mean nearest taxon distance (MNTD; c), functional richness (FRic; d), functional evenness (FEve; e), functional dispersion (FDis, f), functional divergence (FDiv; g).

To control for the confounding effect of species richness, we used a randomization approach to identify regions where phylogenetic functional indices significantly deviated from their expected values. The SES results for the phylogenetic indices showed that the observed values of PD and MNTD were higher than the expected levels from the null model in the southwestern region, including the Hengduan Mountains, a small portion of the Eastern Himalayas, the northern part of South China, as well as the southern parts of Central and East China. These areas exhibited phylogenetic dispersion, with relatively ancient ancestral lineages. In contrast, the northeastern, northern, and northeastern regions of North China, the southern part of Central China, the northern part of South China, and the southeastern Tibetan Plateau showed lower values than expected from the null model, indicating a trend of phylogenetic clustering and younger lineages (Figure 3a,c). Although the distributional pattern of SES MPD largely resembled that of SES PD and SES MNTD, the trend was reversed in the southeastern Tibetan Plateau, southern Hengduan Mountains, and the eastern part of Central China (Figure 3a–c).

The SES of the functional diversity metrics show that, except for Taiwan, the observed values of FRic and FDis in the southeastern regions of China are significantly higher than the expected values based on the null model (Figure 3d,f). In contrast, FEve and FDiv are generally lower than the expected values from the null model in these regions (Figure 3e,g). In the southwestern region, the observed values of FRic, FEve, and FDis are generally lower than the expected values from the random null model (Figure 3d–f), with positive SES values for FDis in the Hengduan Mountains and southern East China, where the observed FDis values exceed the expected values (Figure 3f).

### 3.3 Relationship Between Diversity Metrics and Environmental Variables

**FIGURE 4:**
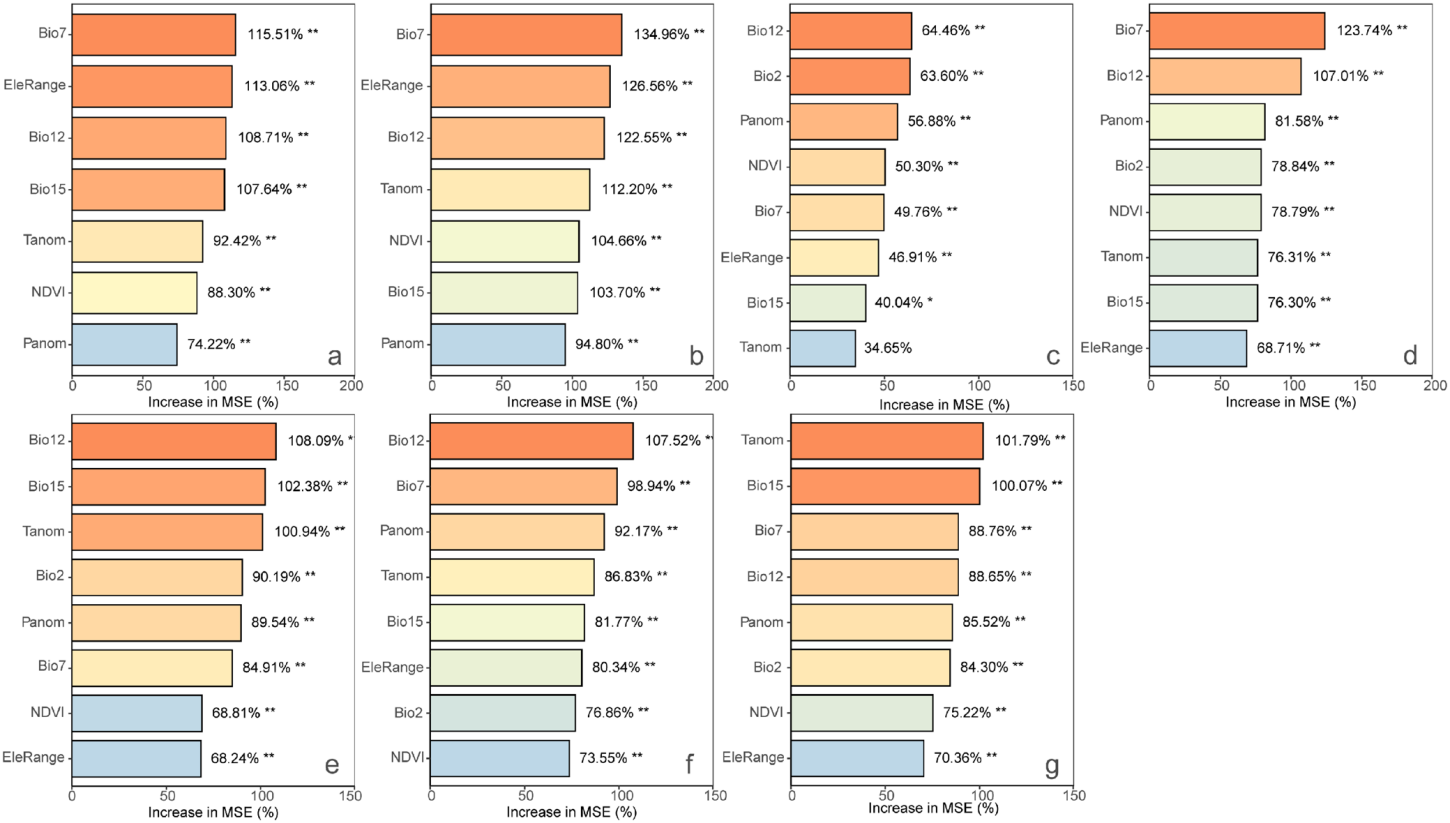
Relative importance of environmental variables to species richness (SR; a), Faith's phylogenetic diversity index (Faith's PD; b), mean pairwise distance (MPD; c), mean nearest taxon distance (MNTD; d), SES of Faith's phylogenetic diversity index (sesPD; e), mean pairwise distance (sesMPD; f), mean nearest taxon distance (sesMNTD; g). *p < 0.05; **p < 0.01.

**FIGURE 5:**
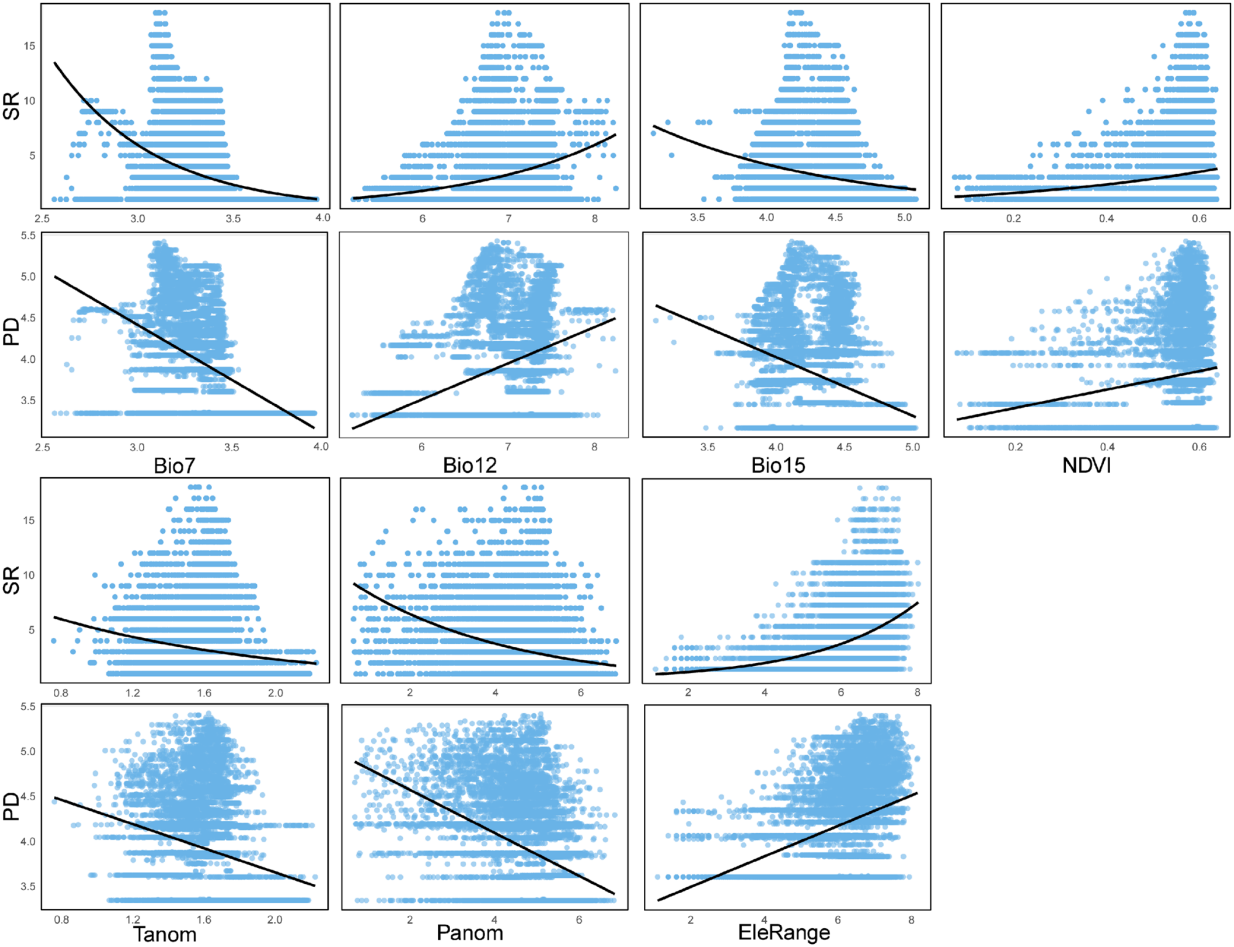
Relationship between the indices and environmental factors.

The random forest analysis indicated that the annual temperature range is the most important factor in explaining the variation in species richness. This variable contributed the most to the increase in MSE in the random forest model, making it the strongest predictor for SR (Figure 4a). Univariate GLM analyses show that as the annual temperature range increases, SR exhibits a decreasing trend (Figure 5). Elevation range also has a significant impact on species richness, showing a positive correlation (Figures 4a, 5).

**FIGURE 6:**
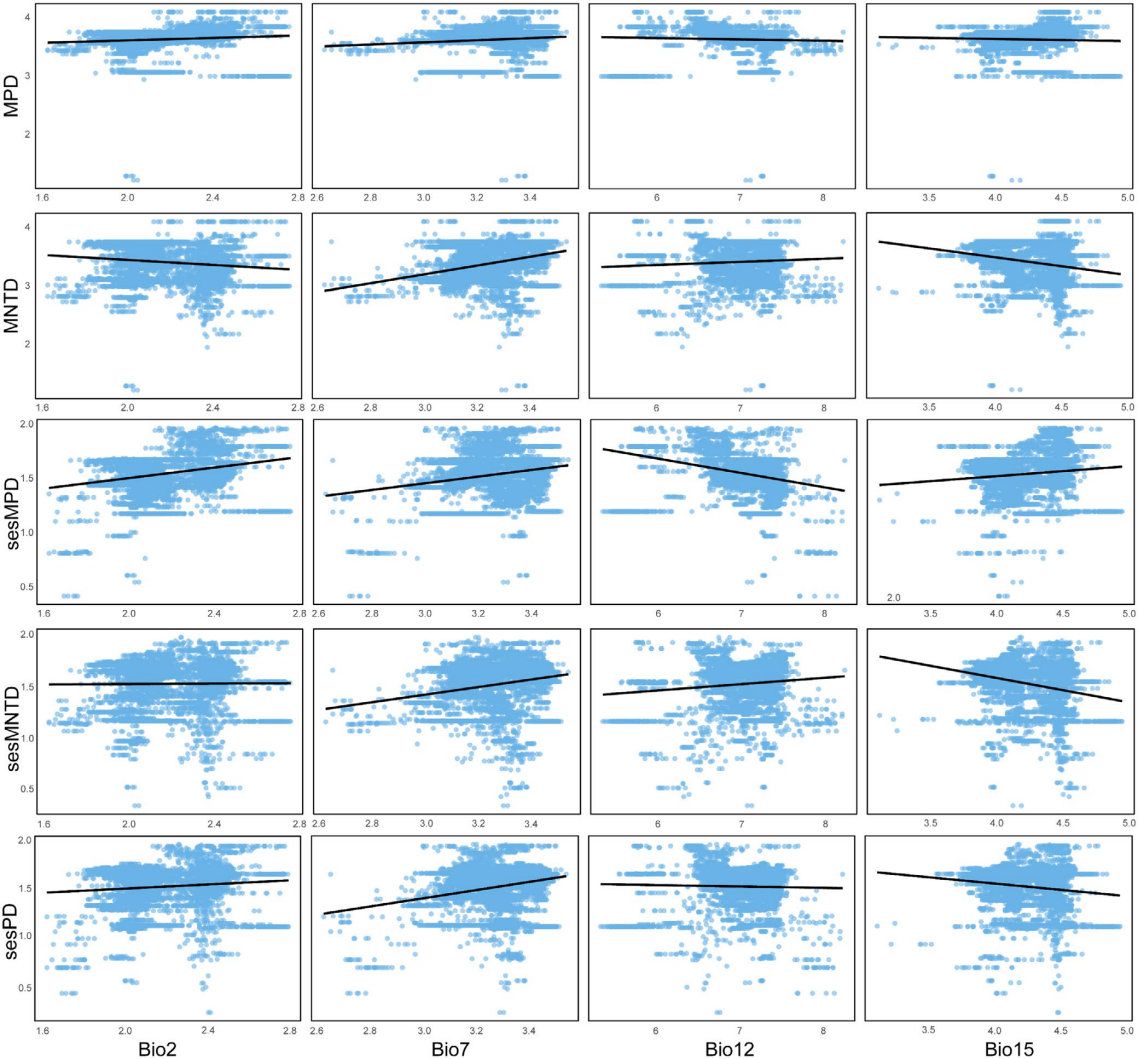
Relationship between the indices and environmental factors.

**FIGURE 7:**
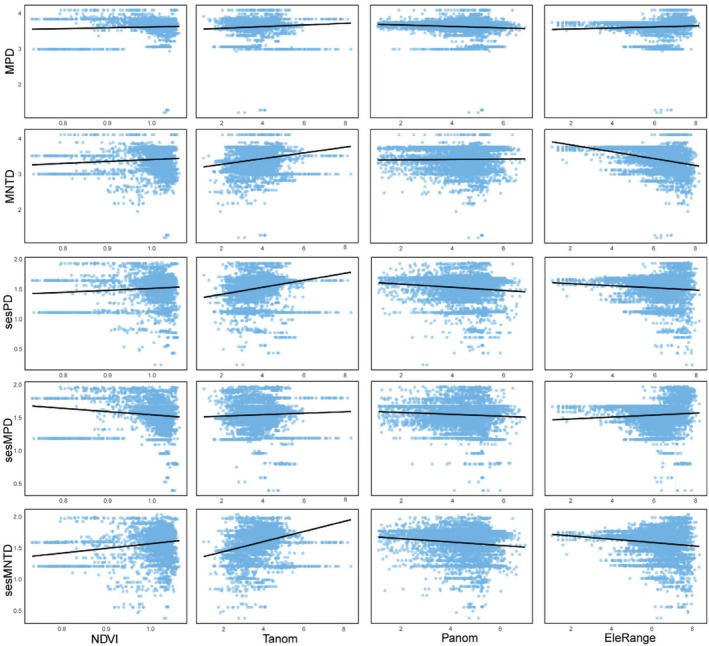
Relationship between the indices and environmental factors.

For phylogenetic diversity metrics, Faith's phylogenetic diversity is mainly influenced by annual temperature range, followed by elevation range, with negative and positive correlations, respectively (Figures 4b, 5). For MPD, sesPD, and sesMPD (Figure 4c,e,f), mean annual precipitation contributes the most to the increase in mean squared error, highlighting its strong predictive importance in these indices, with a negative correlation observed (Figure 6). MAT anomalies significantly affect sesMNTD, with sesMNTD increasing significantly as MAT anomalies rise, demonstrating a positive correlation (Figures 4g, 7).

**FIGURE 8:**
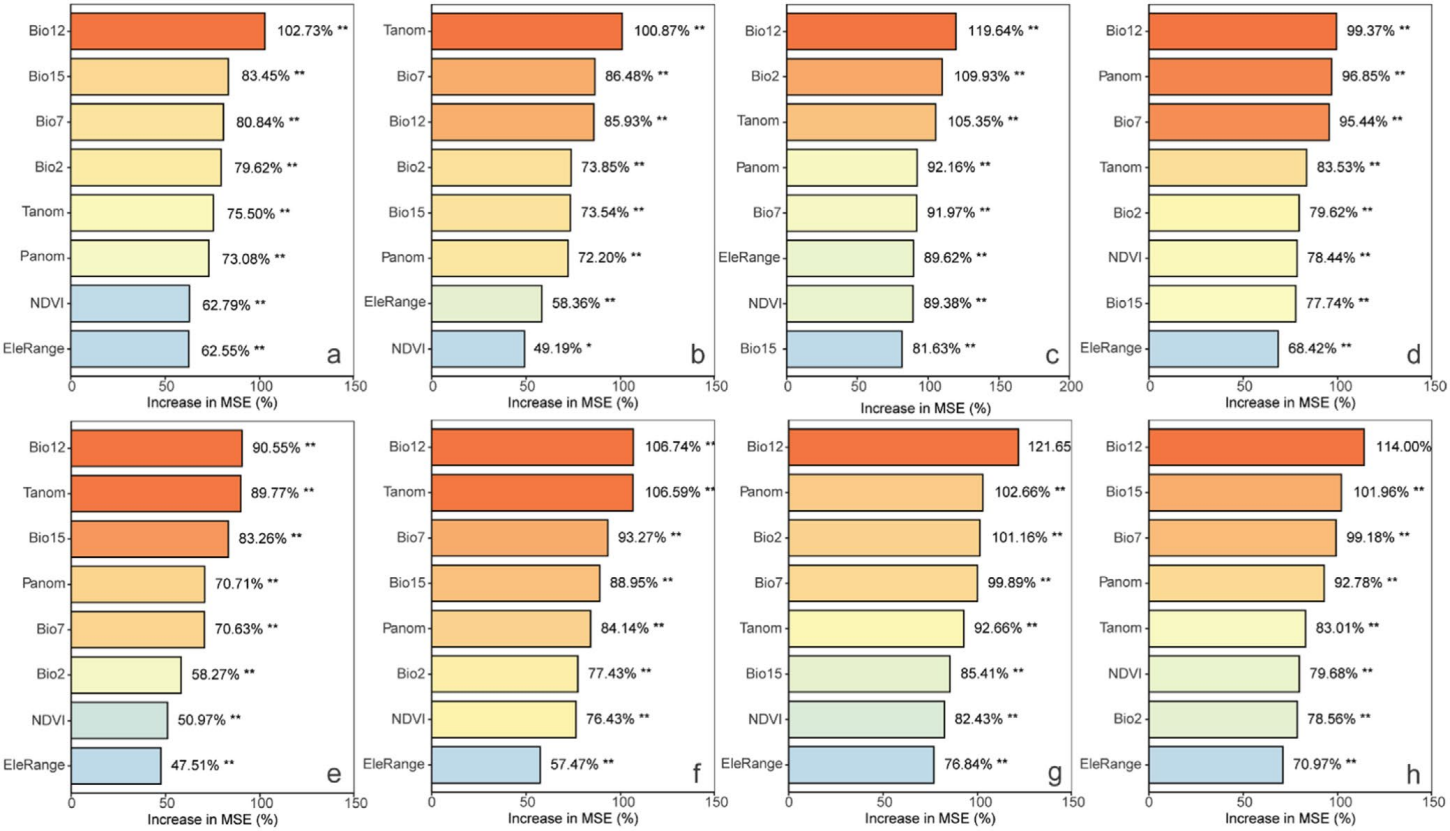
Relative importance of environmental variables to functional richness (FRic; a), functional evenness (FEve; b), functional dispersion (FDis; c), and functional divergence (FDiv; d), SES of functional richness (sesFRic; e), functional evenness (sesFEve; f), functional dispersion (sesFDis; g), and functional divergence (sesFDiv; h). *p < 0.05; **p < 0.01.

**FIGURE 9:**
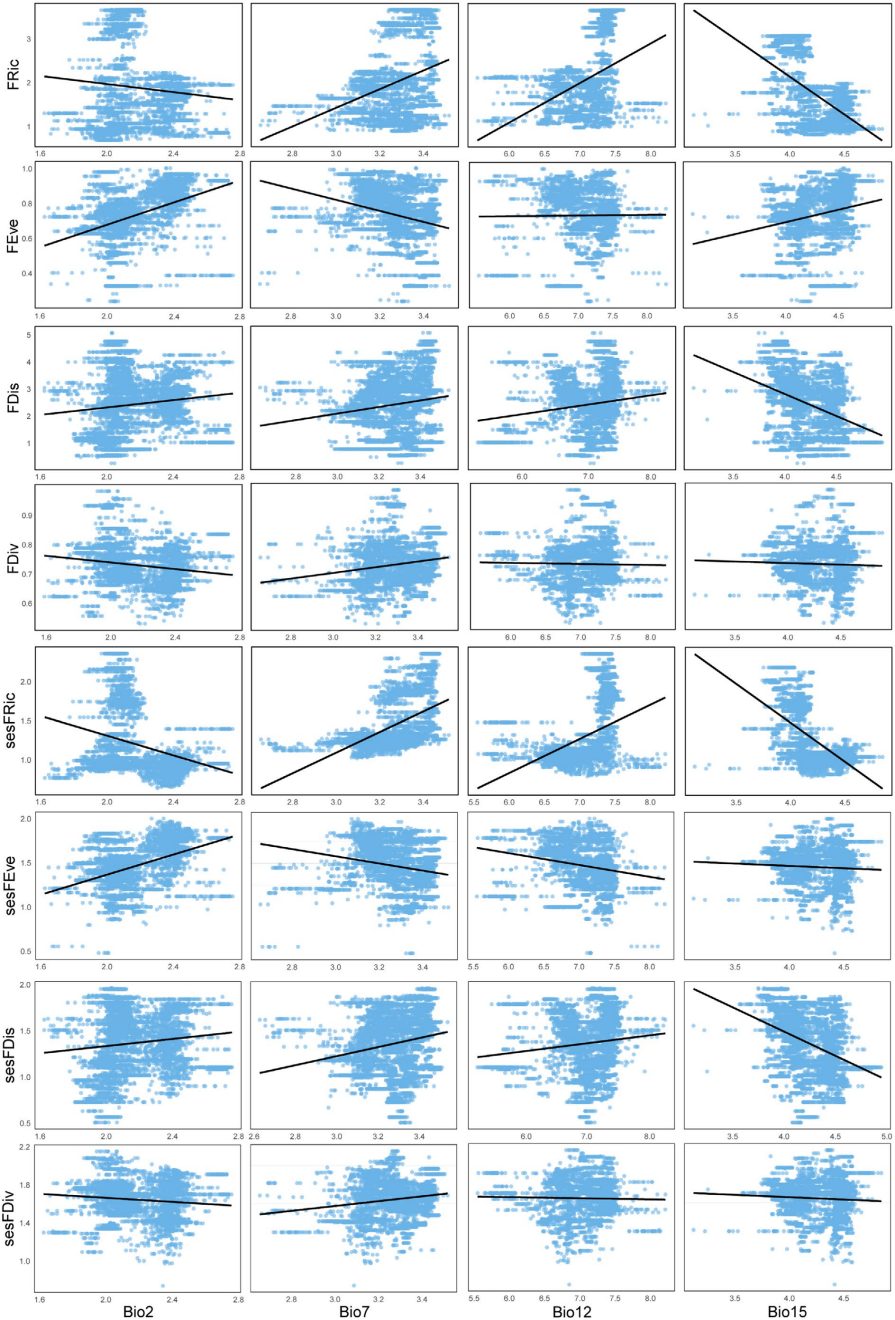
Relationship between the indices and environmental factors.

**FIGURE 10:**
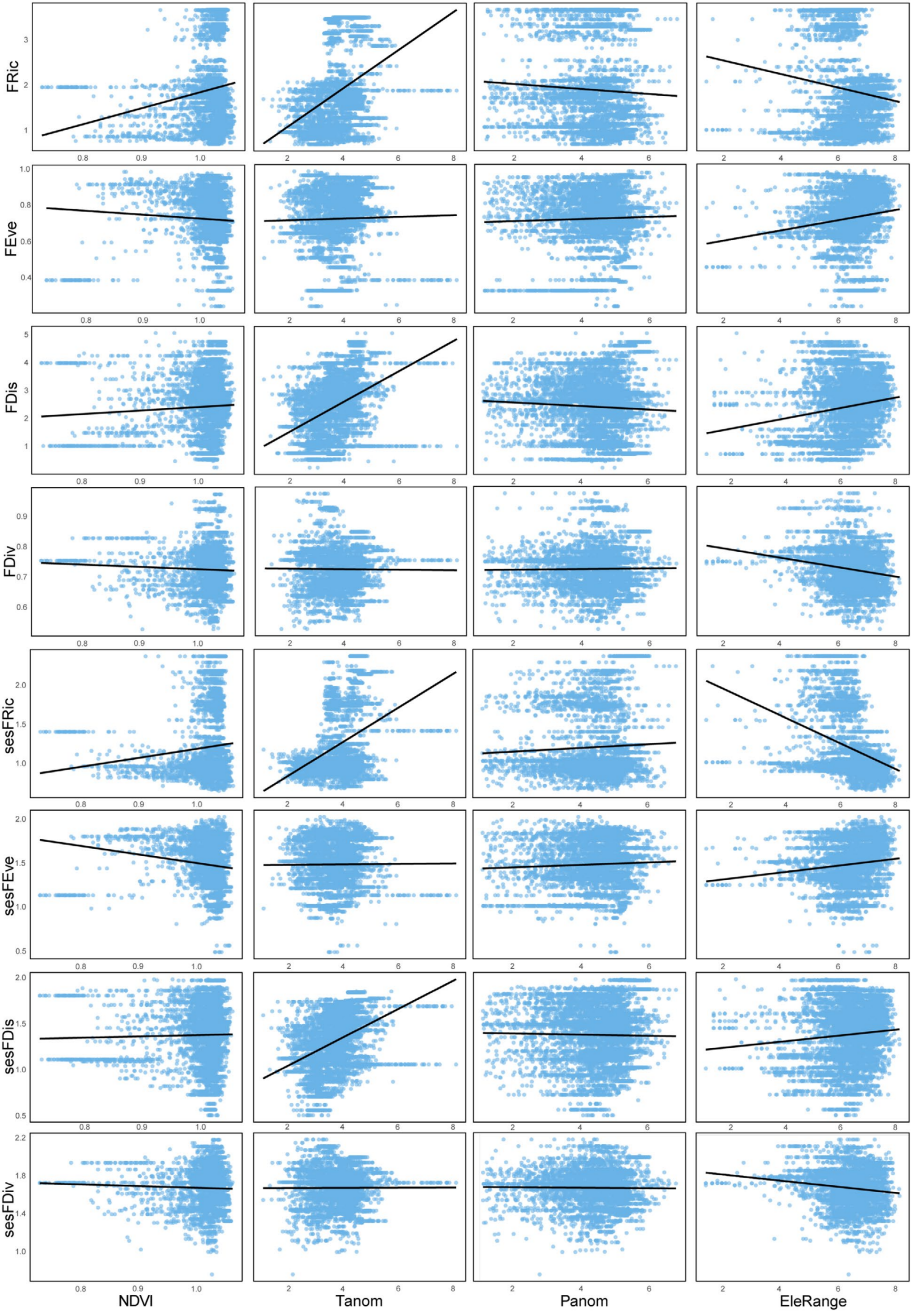
Relationship between the indices and environmental factors.

For functional diversity metrics, mean annual precipitation has higher predictive importance for FRic, FDis, FDiv, sesFRic, sesFEve, sesFDis, and sesFDiv compared to other environmental factors (Figure 8a,c–h). A significant positive correlation is observed between mean annual precipitation and FRic, FDis, sesFRic, and sesFDis, and a negative correlation with FDiv, sesFEve, and sesFDiv (Figure 9). These findings suggest that precipitation limits the co‐occurrence of distantly related species while promoting functional trait differentiation. Furthermore, MAT anomaly significantly predicts FEve and sesFEve, with both metrics increasing in a positive correlation as MAT anomaly rises (Figure 10).

## 4 Discussion

### 4.1 Species Diversity and Its Influencing Factors

The *Lucanus* species in China are predominantly distributed in the southern mountainous regions, particularly in the southwestern mountains and the Wuyi Mountains–Nanling region in the southeast. These areas are primarily located in subtropical climate zones, where the warm and humid conditions, coupled with complex and highly heterogeneous habitats, create an ideal environment for the survival and reproduction of *Lucanus* and other insect species (Harvey et al. 2011). This distributional pattern is consistent with the findings of Fan et al. (2024), who observed similar geographic distributional patterns for Coleopteran insects in their study of insect biodiversity in China. Previous studies on other taxa, such as Hemiptera (Li et al. 2021), angiosperms (Song et al. 2024b), and birds (Liang et al. 2024), have also highlighted the convergence of biodiversity hotspots in the mountainous regions from central to southern China. This similarity underscores the ecological significance of this region as a center of biodiversity. In contrast, regions such as North China, central South China, and Central China generally exhibit lower SR, which is closely associated with the more arid and homogeneous climatic conditions of these areas (Sosa et al. 2018). Furthermore, the flat and uniform terrain of the plains, the absence of microhabitat variation typically found in mountainous regions, and habitat destruction due to agricultural expansion all contribute to the lower SR in these areas (Tamme et al. 2010; Cheng et al. 2023).

Recent studies have demonstrated that temperature, precipitation, and their extreme values throughout the year are widely considered primary environmental factors influencing species diversity and community composition at large spatial scales. Regions with relatively stable climates tend to exhibit higher species richness (Kissling et al. 2011; Heino et al. 2015). As ectothermic organisms, insects are particularly sensitive to fluctuations in external climate conditions, which can affect their body temperature and, in turn, hinder their growth and reproduction (Gruber et al. 2009). Huang (2014) found that climate change could negatively affect the population size of the endemic stag beetle *Lucanus miwai* Kurosawa 1966 in Taiwan. Regions with high topographical heterogeneity, such as the Hengduan Mountains, may promote greater species diversity by offering a range of ecological niches that help mitigate the effects of environmental fluctuations (Marchese 2015). In our study, random forest models and linear regression analysis indicate that stable climate, adequate precipitation, and higher habitat heterogeneity contribute to the survival of *Lucanus*. These factors collectively highlight the critical role of environmental stability and habitat complexity in supporting species persistence, particularly in the context of climate change.

### 4.2 Phylogenetic Diversity, Phylogenetic Structure, and Their Influencing Factors

The distributional pattern of *Lucanus* genus phylogenetic diversity (PD) closely mirrors that of species richness (SR). In regions with relatively low SR, such as South China and central Central China, elevated mean phylogenetic/nearest taxon distances (MPD/MNTD) reveal assemblages of phylogenetically distinct lineages arising from prolonged evolutionary isolation (Zhang et al. 2022). Crucially, high PD‐SR congruence centers coincide with major mountain ranges (e.g., Hengduan and Wuyi‐Nanling), where topographic complexity drives habitat fragmentation, climatic stratification, and niche specialization, collectively fostering both species divergence and the retention of ancient lineages. These findings suggest that the topographic and environmental complexity of mountainous regions is a key driver of the elevated phylogenetic diversity observed in these areas.

In the southwestern mountainous region, especially in the northern part, both phylogenetic overdispersion and clustering coexist, with both ancient and younger lineages present. The oldest lineages are primarily located in the Eastern Himalayas and the Hengduan Mountains, aligning with the evolutionary history of high‐altitude plant communities in this region and the climate‐driven features of these ecosystems (Ding et al. 2020). This phenomenon may be a result of the unique ecological and evolutionary history of the southwestern mountains. As a key area in biodiversity pattern research, the region is home to numerous endemic species and exhibits complex and distinctive evolutionary trajectories (Yu et al. 2020). Mountains, as crucial elements of the ecological framework of the southwestern mountains, play an undeniable role in the evolution of biodiversity. Previous studies have suggested that intense climate changes since the last glacial maximum (LGM) may have significantly impacted the northern regions, leading many species to migrate southward or to higher altitudes. During this period, southern China, experiencing relatively weaker climate fluctuations, became a refuge for species (López‐Pujol et al. 2011a, 2011b; Sandel et al. 2011). The diverse topography in these areas may have provided sufficient ecological stability to buffer the impacts of extreme climate fluctuations.

Notably, southern Tibet presents a combination of high sesMPD (which strongly reflects ancient evolutionary divergence) and low sesMNTD (which similarly indicates ancient evolutionary divergence), suggesting that multiple species groups with large evolutionary distances may coexist in the region. Within these groups, there may also be younger species or those that have undergone rapid radiation and local adaptation (Fan et al. 2024; Qian et al. 2024). This pattern may be related to the climate stability of the Eastern Himalayas during the glacial periods, or species may have found suitable habitats through localized migration, undergoing rapid radiation and adaptive evolution (Lei et al. 2015). In contrast, the Wuyi Mountains–Nanling region in the southeast exhibits low sesMPD and high sesMNTD, indicating that the deep evolutionary lineages of *Lucanus* in this region are relatively few, or their distribution is limited, resulting in lower MPD. However, on more recent evolutionary timescales, certain populations may have undergone regional species radiation, with considerable evolutionary distances between species, thus displaying phylogenetic overdispersion (Fan et al. 2024). This suggests a dynamic evolutionary process in which the region experiences rapid diversification in certain lineages despite its overall lower phylogenetic divergence.

### 4.3 Functional Diversity, Functional Structure, and Their Influencing Factors

Integrating functional trait data provides a powerful strategy for studying the community structure and potential mechanisms of species coexistence in the *Lucanus* genus in China.

Our study found that the functional structure of *Lucanus* in southeastern China exhibits significant functional trait differences, with both FRic and FDis being relatively high. This suggests that species show considerable differentiation in functional traits and have a broad distribution across the functional trait space, which reduces functional overlap. However, the distribution of functional traits in the functional space is not completely uniform, possibly due to the historical adaptation processes of the species. At the same time, both FRic and FDis exceed the expectations of the null model, indicating that *Lucanus* occupies a broader and more dispersed functional space than would be expected from random processes, reflecting significant functional divergence (Laliberté and Legendre 2010; Huang et al. 2022). These characteristics suggest that *Lucanus* species reduce resource competition and promote species coexistence through functional trait differentiation, supporting the applicability of the limiting similarity theory in this region (Mouchet et al. 2010; Aros‐Mualin et al. 2021).

Compared with the southeastern region, the southwestern region exhibits higher FEve, while FRic values are lower than expected from the null model. These results suggest that, with increasing species richness, species tend to occupy the central areas of functional space, leading to tighter niche packing, which implies that the *Lucanus* communities in the southwestern region are significantly influenced by environmental filtering (Kraft et al. 2015; Huang et al. 2022). Additionally, the Hengduan Mountains and the Qinling–Daba Mountains in this region host the oldest ancestral lineages, aligning with the phylogenetic niche conservatism (Wiens and Donoghue 2004). The southwestern region, due to its complex topography, significant elevation gradients, and diverse microclimatic conditions, exhibits high environmental heterogeneity (Yu et al. 2020). The high altitudes, low temperatures, and extreme conditions of mountain gorges promote environmental diversity; but at the same time, due to resource distribution limitations and spatial isolation effects, they significantly increase the survival pressure on species (Manish 2021). The limitations on survival conditions force species to concentrate on adapting to limited resources and specific niches, leading to functional trait convergence in these environments.

This difference is closely linked to the climatic changes in both regions since the last glacial maximum (LGM). Our study found that the increase in annual precipitation and the intensification of climate anomalies have facilitated the expansion and dispersion of functional trait space. This trend highlights the profound impact of enhanced climate change since the LGM on species distribution. More extreme climatic dynamics may have caused the distribution of *Lucanus* species in trait space to become more homogenized and dispersed. This change suggests that, during the LGM, species may have faced stronger interspecific competition pressures or evolved adaptive traits in response to harsh environmental conditions, driving the reshaping of trait differentiation and niche distribution (Mammola et al. 2021; Huang et al. 2023). In contrast to the southwestern mountains, the southeastern region likely experienced more severe climatic changes (Lei et al. 2015), providing a more diverse set of conditions for species trait differentiation, which resulted in higher overall FRic and FDis compared with the southwestern mountains. In the southwestern region, the smaller magnitude of climate change, coupled with the significant influence of environmental filtering, has led to a more concentrated distribution of functional traits within the core of functional space.

### 4.4 Synthesis of Multidimensional Conservation Strategies

The coupled yet spatially asynchronous patterns of species richness, phylogenetic endemism, and functional divergence across southern China's mountain ranges pinpoint the Hengduan and Wuyi–Nanling Mountains as a composite conservation priority. This integrative framework addresses their complementary biogeographic roles: Elevational gradients in these regions preserve microhabitat mosaics buffering climate‐driven range shifts, while cross‐range corridor networks counteract distributional asynchrony among taxa. Divergent conservation targets emerge across spatial scales—stable old‐growth substrates in core Hengduan valleys safeguard relict lineages as evolutionary museums, whereas dynamic disturbance regimes in subtropical peaks sustain contemporary radiation processes (Hilty et al. 2020). Crucially, microhabitat management must align with functional signatures: structural preservation of decaying wood enhances niche complementarity in the Wuyi–Nanling, while habitat filtering conservation dominates in western mountains. This multidimensional strategy amplifies ecosystem resilience by interlacing biodiversity's static repositories and dynamic theaters (Meyer et al. 2015; Birkemoe et al. 2018).

## 5 Conclusion

This study presents the first comprehensive analysis of the genus *Lucanus* in China, revealing its strong montane affinity with distinctive macroecological patterns. Southern China's mountainous systems emerge as dual hotspots, functioning as evolutionary museums preserving ancient lineages and diversification cradles nurturing neo‐endemic clades, particularly in the species‐rich southwest where ancestral lineages persist under significant environmental filtering. Contrastingly, southeastern regions demonstrate exceptional functional richness through trait‐mediated niche partitioning. Notably, we identify critical conservation priorities in areas exhibiting discordant diversity dimensions—regions with low species richness yet high phylogenetic and functional diversity retain irreplaceable evolutionary potential. These multidimensional disjunctions decouple species abundance from both functional dispersion and community evolutionary history, necessitating integrative conservation frameworks that address phylogenetic legacy preservation, functional resilience maintenance, and geographic heterogeneity reconciliation. These differences further emphasize the importance of interpreting biodiversity from multiple dimensions. This study provides new insights into the distributional patterns of *Lucanus* diversity in East Asia and the potential environmental factors influencing these patterns.

## Author Contributions

**Ya Xin Wang:** data curation (equal), formal analysis (equal), investigation (equal), methodology (equal), software (equal), visualization (equal), writing – original draft (equal), writing – review and editing (lead). **Ya Gang Shen:** data curation (equal), formal analysis (equal), investigation (equal), methodology (equal), software (equal), visualization (equal), writing – original draft (equal). **Xue Li Zhu:** data curation (equal), investigation (equal), software (equal). **Xia Wan:** methodology (equal), supervision (lead), writing – review and editing (supporting).

## Ethics Statement

This study complied with the appropriate institutional, national, and international guidelines.

## Conflicts of Interest

The authors declare no conflicts of interest.

## Supporting information


**Figure S1:** Kernel density estimation.

**Figure S2:** Hexagonal binning map.

**Figure S3:** MCMCtree.

**Figure S4:** Annotated diagram of functional traits.

**Table S1:** Specimen measurements, behavioral characteristics, and functional traits of Lucanus species analyzed in this study.
**Table S2:** Filtered environmental variables and their abbreviations used in the analysis of Lucanus diversity.

**Table S3:** AUC values and classification thresholds of the MaxEnt model.

**Table S4:** Correlation coefficients of environmental factors (extracted by grid cells).

**Data S1:** ece371954‐sup‐0008‐DataS1.xlsx.

## Data Availability

Species distributional data used in this study are deposited in Mendele (https://data.mendeley.com/datasets/yp5f7wjng7/1); functional trait data are in Supporting Information. GenBank: PV541516‐PV541572, PV560615‐PV560661, PV565519‐PV565526.
