# Supplementary figures and images for: Multifaceted Biodiversity Patterns and Influencing Factors of Lucanus Stag Beetles (Coleoptera, Lucanidae) in China
Source: Ecol Evol. 2025 Aug 12;15(8):e71954. doi: 10.1002/ece3.71954 (PMC12340431; doi:10.1002/ece3.71954)

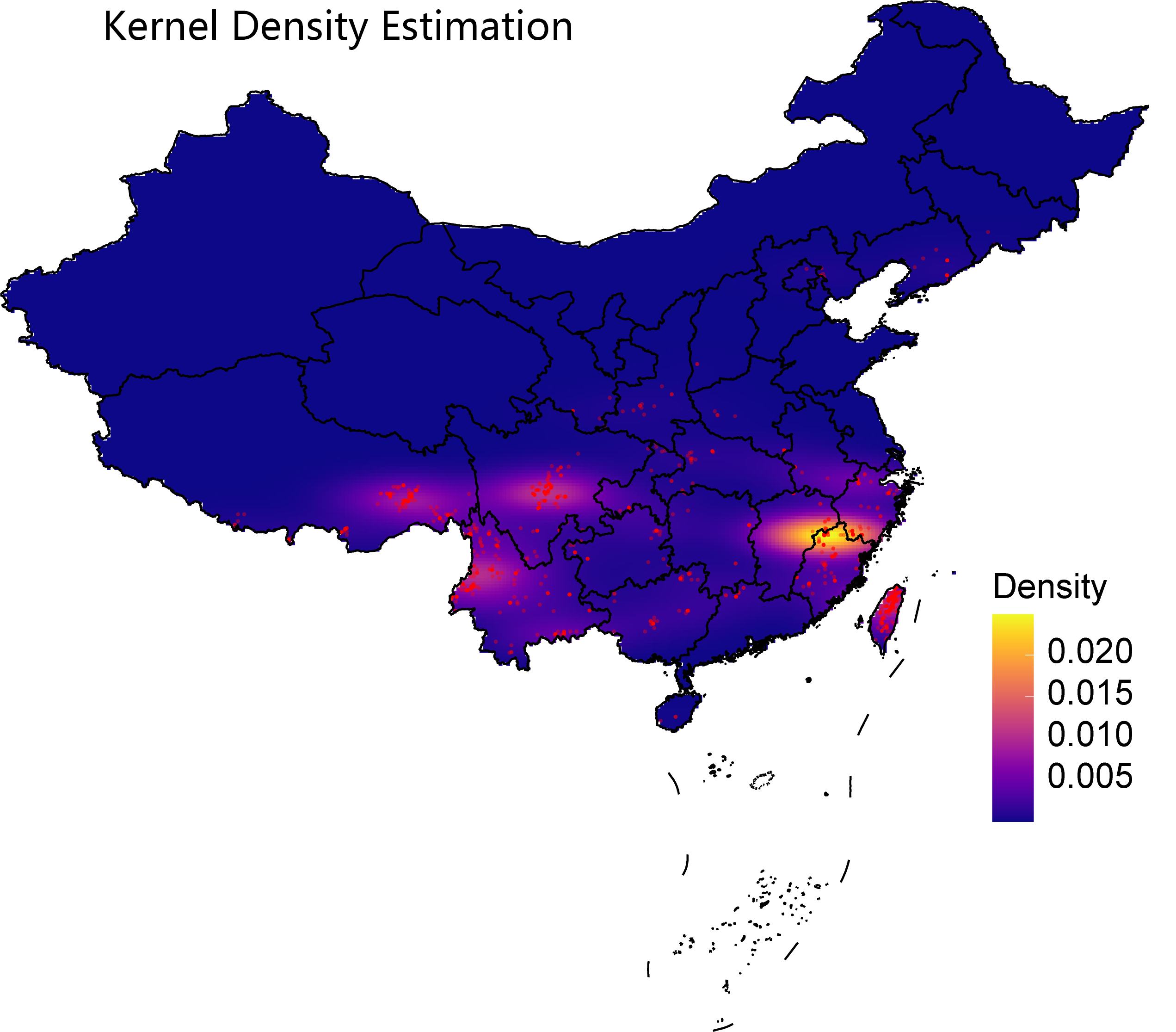

Supplement: Supplementary file 1 — Figure S1: Kernel density estimation. [file ECE3-15-e71954-s006.tif]

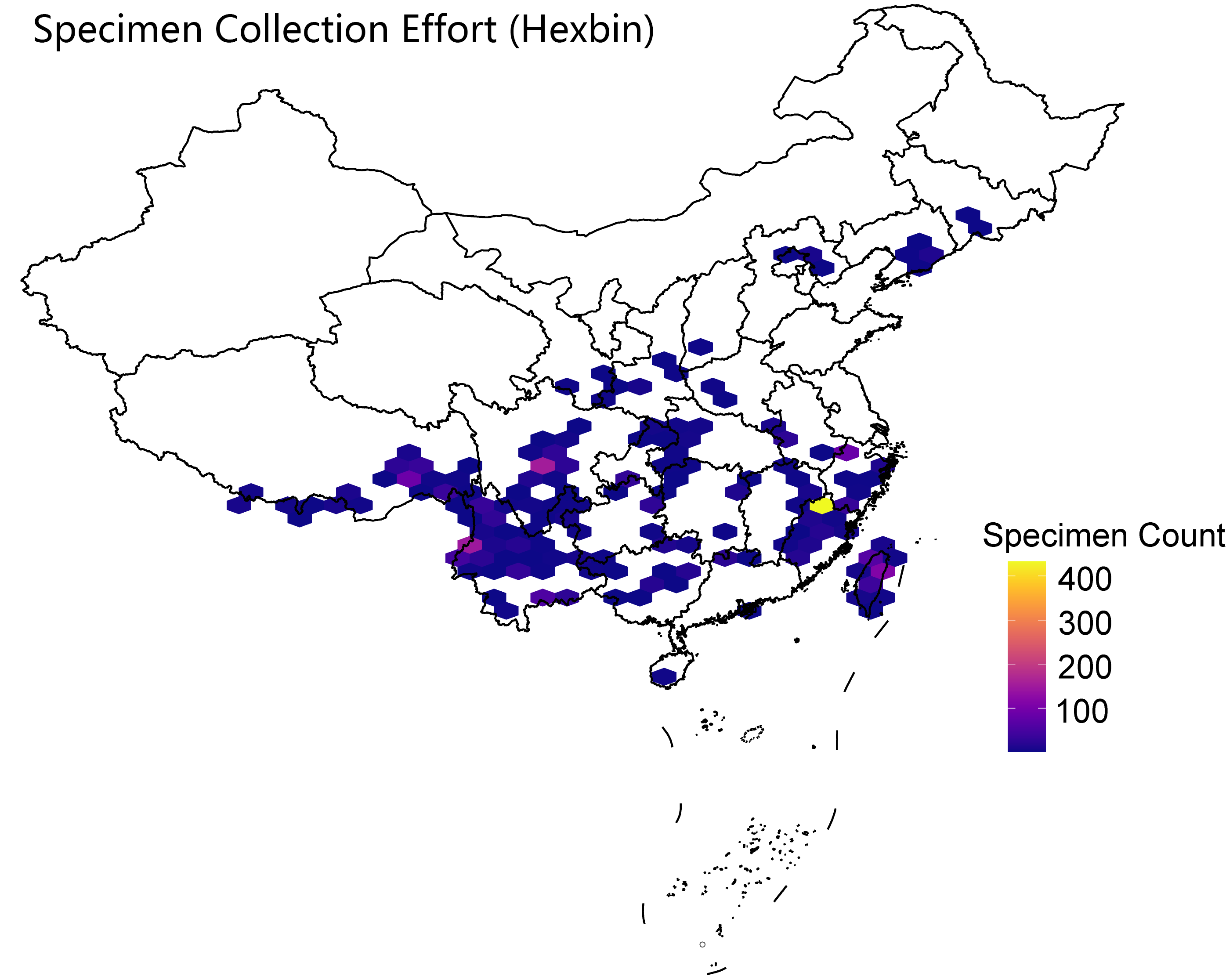

Supplement: Supplementary file 2 — Figure S2: Hexagonal binning map. [file ECE3-15-e71954-s003.tif]

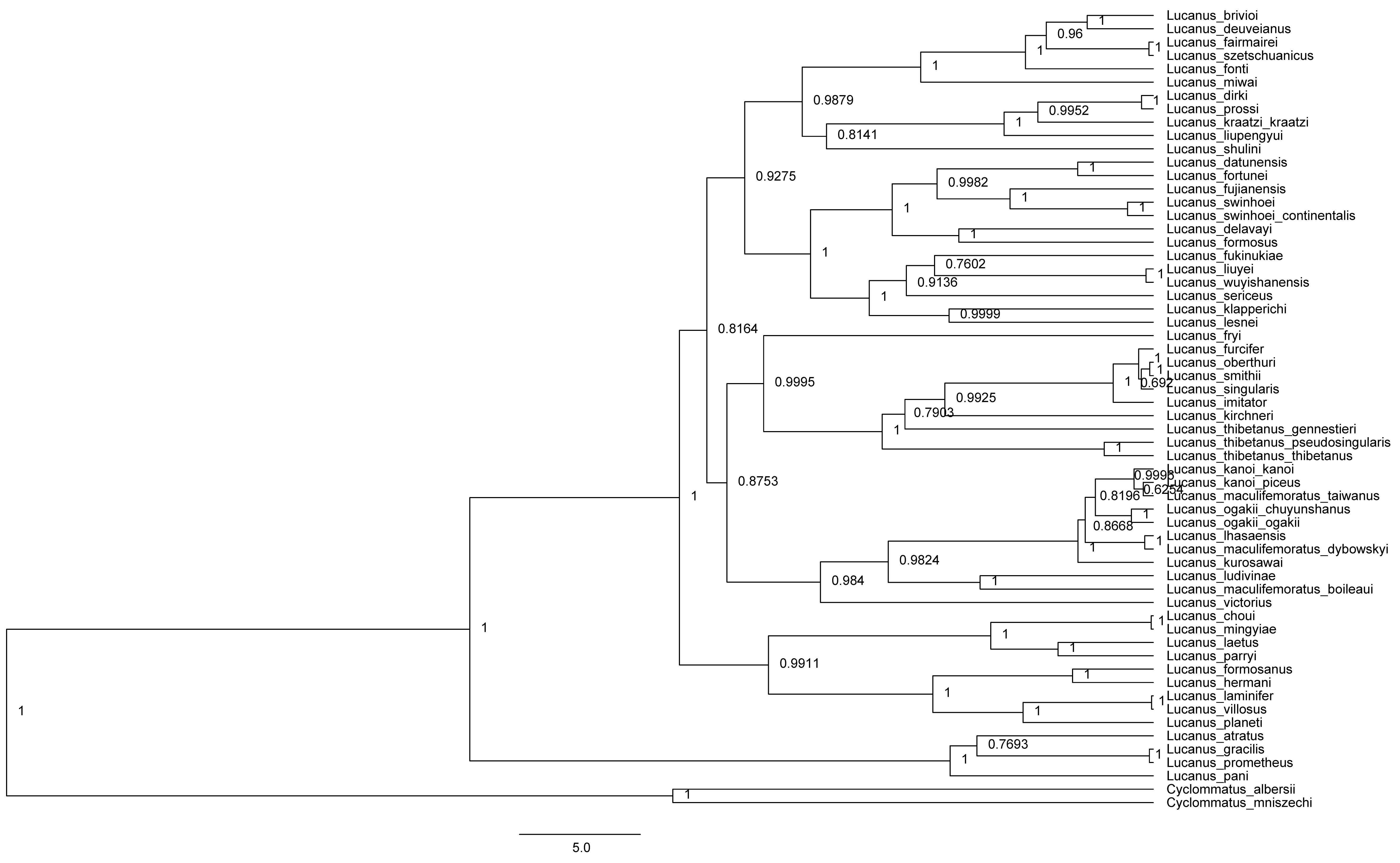

Supplement: Supplementary file 3 — Figure S3: MCMCtree. [file ECE3-15-e71954-s002.tif]

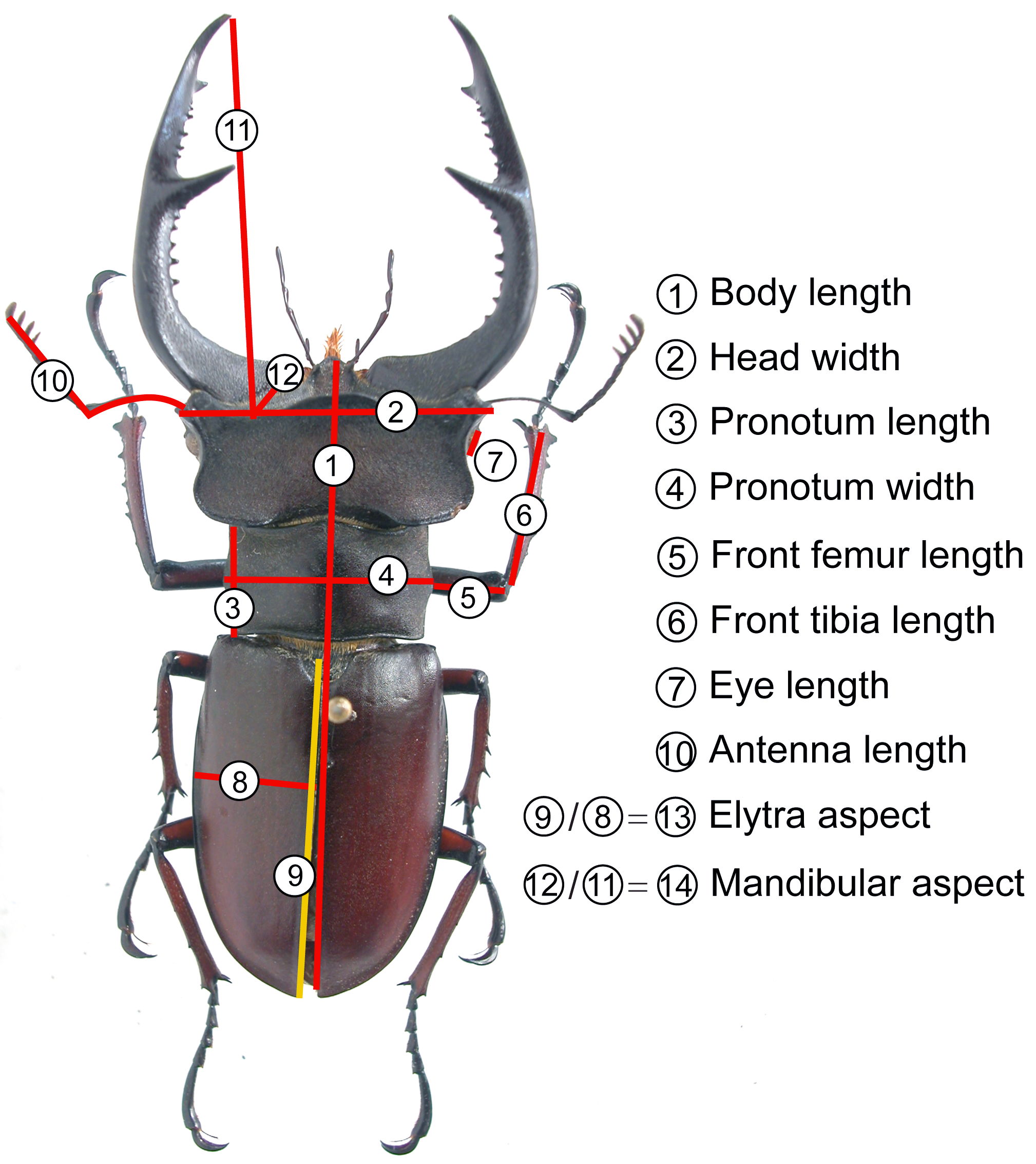

Supplement: Supplementary file 4 — Figure S4: Annotated diagram of functional traits. [file ECE3-15-e71954-s005.tif]
