# Supplementary material for: Multifaceted Biodiversity Patterns and Influencing Factors of Lucanus Stag Beetles (Coleoptera, Lucanidae) in China
Source: Ecol Evol. 2025 Aug 12;15(8):e71954. doi: 10.1002/ece3.71954 (PMC12340431; doi:10.1002/ece3.71954)
Supplement: Supplementary file 5 — Table S1: Specimen measurements, behavioral characteristics, and functional traits of Lucanus species analyzed in this study. Table S2: Filtered environmental variables and their abbreviations used in the analysis of Lucanus diversity. [file ECE3-15-e71954-s008.docx]

|  | Morphological  trait | functions of the  trait |
| --- | --- | --- |
| Body shape | Body length | Fundamental trait in the species  biology, dispersal ability,  oxygen transport, metabolic rate  (Den Boer, 1990; Kaiser et al., 2007; Peters, 1983) |
|  | Head width | Prey size and foraging speed  (Kaspari & Weiser, 1999) |
|  | Pronotum length and width | Relative robustness (Fountain-Jones et al., 2015; Hagge et al., 2021) |
| Locomotion | Elytra aspect ratio | Flight performance (manoeuvrability, speed, Distance)  (Hassall, 2015; Wootton, 1992) |
|  | Front femur length | Dispersal ability, resource acquisition, foraging efficiency ands urface temperature  (Hurlbert et al., 2008; Teuscher et al., 2009; Krasnov et al., 1996) |
|  | Front tibia length | Dispersal ability, resource acquisition, foraging efficiency ands urface temperature  (Hurlbert et al., 2008; Teuscher et al., 2009; Krasnov et al., 1996) |
| Sensory | Antenna length | Olfactory communication; diurnal or nocturnal activity  (Elgar et al., 2018; Ribera et al., 1999) |
|  | Eye length | Diurnal or nocturnal activity and  microhabitat use  (Talarico et al., 2011; Talarico et al., 2007) |
| Foraging | Mandibular aspect ratio | Reproductive mating ; fighting  (Bai et al., 2015; Goyens et al., 2016; Mills et al., 2016) |

**Appendix table 1 Specimen measurements, behavioral characteristics and functional traits of *Lucanus* species analyzed in this study**

Den Boer, P. J. (1990). The survival value of dispersal in terrestrial arthropods. *Biological Conservation, 54*(3), 175-192.

Kaiser, A., Klok, C. J., Socha, J. J., Lee, W. K., Quinlan, M. C., & Harrison, J. F. (2007). Increase in tracheal investment with beetle size supports hypothesis of oxygen limitation on insect gigantism. *Proceedings of the National Academy of Sciences, 104*(32), 13198-13203.

Peters, R. H. (1986). *The ecological implications of body size*. Cambridge university press.

Fountain-Jones, N. M., Baker, S. C., & Jordan, G. J. (2015). Moving beyond the guild concept: developing a practical functional trait framework for terrestrial beetles. *Ecological Entomology, 40*(1), 1-13.

Hagge, J., Müller, J., Birkemoe, T., Buse, J., Gossner, M. M., Gruppe, A., ... & Drag, L. (2021). What does a threatened saproxylic beetle look like? Modelling extinction risk using a new morphological trait database. *The Journal of Animal Ecology, 90*(8), 1934-1947.

Hassall, C. (2015). Strong geographical variation in wing aspect ratio of a damselfly, Calopteryx maculata (Odonata: Zygoptera). *PeerJ, 3*, e1219.

Wootton, R. (1992). Functional morphology of insect wings. *Annual Review of Entomology, 37*(1), 113–140.

Hurlbert, A. H., Ballantyne, F., & Powell, S. (2008). Shaking a leg and hot to trot: the effects of body size and temperature on running speed in ants. *Ecological Entomology, 33*(1).

Teuscher, M., Braendle, M., Traxel, V., & Brandl, R. (2009). Allometry between leg and body length of insects: lack of support for the size–grain hypothesis. *Ecological Entomology, 34*(6), 718-724.

Krasnov, B., Ward, D., & Shenbrot, G. (1996). Body size and leg length variation in several species of darkling beetles (Coleoptera: Tenebrionidae) along a rainfall and altitudinal gradient in the Negev Desert (Israel). *Journal of Arid Environments*, *34*(4), 477-490.

Elgar, M. A., Zhang, D., Wang, Q., Wittwer, B., Pham, H. T., Johnson, T. L., ... & Coquilleau, M. (2018). Insect antennal morphology: the evolution of diverse solutions to odorant perception. *The Yale journal of biology and medicine*, *91*(4), 457.

Ribera, I., Foster, G. N., Downie, I. S., McCracken, D. I., & Abernethy, V. J. (1999). A comparative study of the morphology and life traits of Scottish ground beetles (Coleoptera, Carabidae). *Annales Zoologici Fennici, 36*(1), 21–37.

Talarico, F., Brandmayr, P., Giglio, A., Massolo, A., & Brandmayr, T. Z. (2011). Morphometry of eyes, antennae and wings in three species of Siagona (Coleoptera, Carabidae). *ZooKeys*, (100), 203.

Talarico, F., Romeo, M., Massolo, A., Brandmayr, P., & Zetto, T. (2007). Morphometry and eye morphology in three species of Carabus (Coleoptera: Carabidae) in relation to habitat demands. *Journal of Zoological Systematics and Evolutionary Research*, *45*(1), 33-38.

Bai, M., Li, S., Lu, Y., Yang, H., Tong, Y., & Yang, X. (2015). Mandible evolution in the Scarabaeinae (Coleoptera: Scarabaeidae) and adaptations to coprophagous habits. *Frontiers in Zoology*, *12*(1), 30.

Goyens, J., Dirckx, J., & Aerts, P. (2016). Jaw morphology and fighting forces in stag beetles. *Journal of Experimental Biology*, *219*(18), 2955-2961.

Mills, M. R., Nemri, R. S., Carlson, E. A., Wilde, W., Gotoh, H., Lavine, L. C., & Swanson, B. O. (2016). Functional mechanics of beetle mandibles: honest signaling in a sexually selected system. *Journal of Experimental Zoology Part A: Ecological Genetics and Physiology*, *325*(1), 3-12.

**Appendix table 2 Filtered environmental variables and their abbreviations used in the analysis of *Lucanus* diversity**

| Types | Environmental Variable | Abbreviation |
| --- | --- | --- |
| Contemporary Climate Variables | Mean Diurnal Temperature Range | Bio2 |
|  | Annual Temperature Range | Bio7 |
|  | Mean Annual Precipitation | Bio12 |
|  | Precipitation Variance | Bio15 |
| Vegetation Factors | Normalized Difference Vegetation Index | NDVI |
| Paleoclimate Fluctuation | Atemperature Anomaly | Tanom |
|  | Precipitation Anomaly | Panom |
| Habitat Heterogeneity | Elevation range | EleRange |
